# Association Between Early-Pregnancy Mean Arterial Pressure and Onset Timing of Hypertensive Disorders of Pregnancy: A Retrospective Cohort Study in Japan

**DOI:** 10.7759/cureus.112389

**Published:** 2026-07-10

**Authors:** Kazuma Tagami, Hiromi Himuro, Ayuko Hirayama, Tetsuro Hoshiai, Takeo Otsuki, Masatoshi Saito

**Affiliations:** 1 Obstetrics, Tohoku University Hospital, Sendai, JPN; 2 Obstetrics and Gynecology, Sendai City Hospital, Sendai, JPN

**Keywords:** hypertensive disorders of pregnancy (hdp), japanese population, mean arterial pressure, multinomial logistic regression, retrospective cohort

## Abstract

Background

Mean arterial pressure (MAP) in early pregnancy is a predictor of hypertensive disorders of pregnancy (HDP). However, no studies in Japanese populations have examined the association between MAP and HDP stratified by gestational age at onset. This study aimed to investigate the association between MAP in early pregnancy and gestational age-specific HDP subtypes in a Japanese population.

Methods

This study included 2,030 Japanese pregnant women. Multinomial logistic regression models were used to investigate the association between MAP in early pregnancy and gestational age-specific HDP subtypes (early-onset, preterm late-onset, and term late-onset).

Results

Higher MAP in early pregnancy was significantly associated with an increased risk of HDP across all gestational age-specific subtypes. The adjusted odds ratios (95% confidence intervals (CIs)) for early-onset HDP were 1.147 (95% CI: 1.102-1.194), preterm late-onset HDP were 1.105 (95% CI: 1.067-1.145), and term late-onset HDP were 1.077 (95% CI: 1.051-1.105).

Conclusions

Higher MAP in early pregnancy was associated with an increased risk of HDP, with a stronger association observed for earlier onset.

## Introduction

Hypertensive disorders of pregnancy (HDP) are common complications associated with adverse perinatal outcomes and an increased risk of non-communicable diseases later in life for both mothers and their offspring [1,2]. HDP is classified by gestational age at onset into early-onset (<34 weeks) and late-onset (≥34 weeks) subtypes, with early-onset HDP associated with poorer outcomes [3]. In addition, delivery intervention for late preterm HDP (34-36 weeks) may reduce maternal complications, including eclampsia, but can increase neonatal risks such as respiratory morbidity [4,5]. In preeclampsia, a subtype of HDP, longer disease duration has been associated with worse long-term maternal cardiovascular outcomes [6]. Moreover, low-dose aspirin has been shown to reduce the risk of preterm-onset disease [3]. Therefore, further subclassification of late-onset HDP into preterm and term categories may have clinical implications. Mean arterial pressure (MAP) reflects maternal systemic hemodynamics and contributes to the regulation of organ perfusion [7,8]. MAP has been associated with the development of preeclampsia [8-11].

Although FMF/ASPRE-based prediction models have demonstrated high accuracy for preeclampsia screening, their implementation in routine clinical practice is not yet universal, partly due to the requirement for specialized measurements such as uterine artery Doppler and placental biomarkers (e.g., placental growth factor). Moreover, first-trimester MAP has been reported to have a stronger association with early-onset than with late-onset preeclampsia; however, evidence in Japanese cohorts remains limited [11]. Furthermore, no studies have specifically examined the association between MAP in early pregnancy and HDP when stratified into early-onset, preterm late-onset, and term late-onset subtypes, especially in Japanese populations.

This study aimed to investigate the association between MAP in early pregnancy and HDP, which were classified into early-onset (20 to <34 weeks), preterm late-onset (34 to <37 weeks), and term late-onset (≥37 weeks).

## Materials and methods

Study design

**Figure 1:**
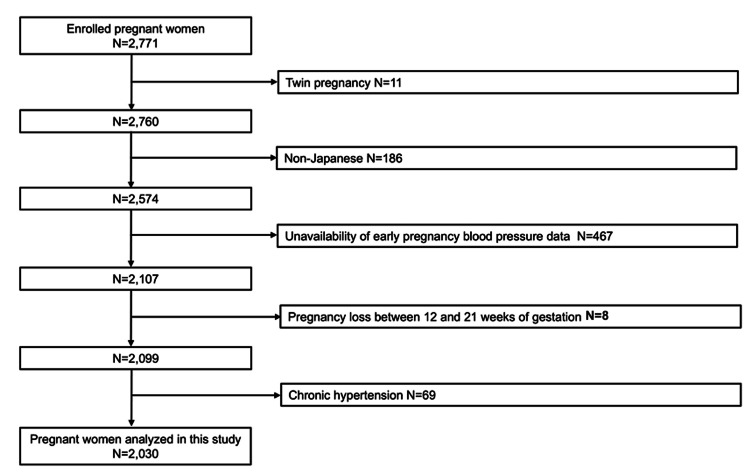
Flowchart of this study

This retrospective cohort study included women who delivered at Sendai City Hospital, Japan, between January 2023 and December 2025. This study was approved by the Ethics Committee of Sendai City Hospital (IRB 20260107). All maternal information was collected from medical records. A total of 2,771 pregnant women were enrolled in this study. A flowchart of participant selection is shown in Figure 1. Participants were excluded for the following reasons: twin pregnancy (N=11), non-Japanese (N=186), unavailability of early pregnancy blood pressure data (N=467), pregnancy loss between 12 and 21 weeks of gestation (N=8), and chronic hypertension (including HDP diagnosed before 20 weeks of gestation) according to the Japanese Society for the Study of Hypertension in Pregnancy classification (N=69) [3]. Finally, 2,030 pregnant women were included in this study.

Exposure

MAP in early pregnancy was calculated using the standard formula, DBP + (SBP − DBP) / 3, based on a single blood pressure measurement obtained between 10 and 13 weeks of gestation at routine first-trimester prenatal visits in our institution. Blood pressure was measured in the seated position after an appropriate rest period according to a standardized institutional protocol. Measurements were primarily performed using automated sphygmomanometers; however, manual measurements were occasionally used in clinical practice when necessary. No distinction between device types was recorded in the present analysis. MAP in early pregnancy was treated as a continuous variable and analyzed per 1-mmHg increase as the primary exposure [12]. As a supplementary sensitivity analysis, MAP in early pregnancy was categorized into two groups: <90 mmHg and ≥90 mmHg, based on previous systematic reviews and meta-analyses [10].

Outcome

HDP was defined as systolic blood pressure (BP) ≥ 140 mmHg and/or diastolic BP ≥ 90 mmHg occurring at ≥ 20 weeks of gestation [3]. Furthermore, HDP was categorized into three subtypes based on the gestational age at diagnosis: early-onset HDP (diagnosed from 20 to < 34 weeks of gestation), preterm late-onset HDP (diagnosed from 34 to < 37 weeks of gestation), and term late-onset HDP (diagnosed at ≥ 37 weeks of gestation) [13,14].

Other variables

Other variables included early pregnancy body mass index (BMI), maternal age, conception method, family history of hypertension, and parity. Early pregnancy BMI was calculated as body weight (kg) divided by the square of height (m²), and participants were categorized into two groups: <25 and ≥25 kg/m². Maternal age was categorized as <35 and ≥35 years. Conception methods were classified into three categories: spontaneous conception, non-ART (i.e., ovulation induction, intrauterine insemination, and timed intercourse), and ART. A family history of hypertension was defined as hypertension in either parent. Parity was categorized as primipara and multipara. A history of HDP was categorized as present or absent.

Statistical analysis

A multinomial logistic regression model was employed to investigate the association between MAP in early pregnancy and HDP. In the supplementary sensitivity analysis, a MAP in early pregnancy of <90 mmHg was set as the reference category. All categorical variables are expressed as numbers (percentages). Model 1 was the crude model. Model 2 was adjusted for confounding factors, including maternal age, conception methods, family history of hypertension, parity, and history of HDP. Model 3 was additionally adjusted for early pregnancy BMI as a potentially modifiable factor. Multiple imputation by chained equations was performed to address missing data for several covariates after confirming that no strong multicollinearity was present. The procedure for multiple imputation was as follows: 20 datasets were created and analyzed using the same model. After these results were combined, the pooled estimates were reported as the adjusted model. R software version 4.5.1 (The R Foundation, Vienna, Austria) was used for all statistical analyses.

## Results

Characteristics

The maternal characteristics are shown in Table 1. Among 2,030 pregnant women in this study, 268 (13.2%) had MAP≥90 mmHg in early pregnancy. The number of pregnant women according to the HDP category was as follows: early-onset HDP (N = 49; 2.4%), preterm late-onset HDP (N = 55; 2.5%), and term late-onset HDP (N = 104; 5.1%). Among pregnant women with MAP ≥90 mmHg in early pregnancy, the numbers of early-onset HDP (N = 25; 9.3%), preterm late-onset HDP (N = 20; 7.4%), and term late-onset HDP (N = 29; 10.7%) were observed.

**Table 1:** Maternal characteristics ^1^ Continuous variables are expressed as mean (SD). Categorical variables are expressed as N (%). Abbreviations: ART, assisted reproductive technology; BMI, body mass index; HDP, hypertensive disorders of pregnancy; MAP, mean arterial pressure.

Characteristic	N = 2,030^1^
Maternal age, N (%)	
<35 years	1,339 (66.0%)
≥35 years	691 (34.0%)
Family history of hypertension, N (%)	486 (27.7%)
Missing	275
Parity, N (%)	
Primipara	1,098 (54.1%)
Multipara	932 (45.9%)
History of HDP, N (%)	66 (3.3%)
Pre-pregnancy BMI, N (%)	
<25 kg/m^2^	1,609 (84.5%)
≥25 kg/m^2^	296 (15.5%)
Missing	125
Conception method, N (%)	
Spontaneous pregnancy	1,617 (79.7%)
Non-ART	124 (6.1%)
ART	289 (14.2%)
MAP in early pregnancy (mmHg)	79.1 ± 9.1
Gestational weeks at delivery, weeks	38.9 ± 1.6
HDP, N (%)	
Early-onset HDP	49 (2.4%)
Preterm late-onset HDP	55 (2.7%)
Term late-onset HDP	102 (5.0%)

Associations between MAP in early pregnancy and early-onset, preterm late-onset, and term late-onset HDP are illustrated in Table 2. Higher MAP in early pregnancy was significantly associated with an increased risk of HDP across all subtypes in crude and adjusted models (p-values <0.001). For early-onset HDP, MAP in early pregnancy was significantly associated in Model 2 (adjusted OR 1.157 per 1 mmHg increase, 95% confidence interval (CI): 1.113-1.202) and Model 3 (adjusted OR 1.145, 95% CI: 1.100-1.192). Similarly, MAP was associated with preterm late-onset HDP in Model 2 (adjusted OR 1.105, 95% CI: 1.067-1.144) and Model 3 (adjusted OR 1.104, 95% CI: 1.066-1.144), and term late-onset HDP in Model 2 (adjusted OR 1.078, 95% CI: 1.051-1.105) and Model 3 (adjusted OR 1.075, 95% CI: 1.048-1.103), although the magnitude of association tended to decrease with increasing gestational age at onset. In additional analyses, pregnant women with MAP in early pregnancy ≥90 mmHg had a significantly higher risk of early-onset HDP in Model 2 (adjusted OR 7.759, 95% CI: 4.290-14.036) and Model 3 (adjusted OR 6.319, 95% CI: 3.430-11.642), preterm late-onset HDP in Model 2 (adjusted OR 4.030, 95% CI: 2.248-7.225) and Model 3 (adjusted OR 3.860, 95% CI: 2.126-7.009), and term late-onset HDP in Model 2 (adjusted OR 2.898, 95% CI: 1.810-4.641) and Model 3 (adjusted OR 2.686, 95% CI: 1.661-4.344), compared with those with MAP <90 mmHg.

**Table 2:** Associations between MAP in early pregnancy and early-onset, preterm late-onset, and term late-onset HDP Model 1: Crude model. Model 2: Adjusted for maternal age, conception methods, family history of hypertension, parity, and history of HDP. Model 3: Adjusted for pre-pregnancy BMI in addition to Model 2. Abbreviations: BMI, body mass index; HDP, hypertensive disorders of pregnancy; MAP, mean arterial pressure.

Outcome	P-value (MAP as a continuous variable)	Continuous variable, per 1 mmHg of MAP increase	MAP <90 mmHg, N = 1,762	MAP ≥90 mmHg, N = 268
Early-onset HDP, N (%)	NA	NA	24 (1.4)	25 (9.3)
Model 1: Crude OR (95% Cl)	< 0.001	1.163 (1.119–1.208)	Reference	8.702 (4.875–15.534)
Model 2: Crude OR (95% Cl)	< 0.001	1.157 (1.113–1.202)	Reference	7.759 (4.290–14.036)
Model 3: Crude OR (95% Cl)	< 0.001	1.145 (1.100–1.192)	Reference	6.319 (3.430–11.642)
Preterm late-onset HDP, N (%)	NA	NA	35 (2.0)	20 (7.3)
Model 1: Crude OR (95% Cl)	< 0.001	1.116 (1.079–1.154)	Reference	4.774 (2.702–8.434)
Model 2: Crude OR (95% Cl)	< 0.001	1.105 (1.067–1.144)	Reference	4.030 (2.248–7.225)
Model 3: Crude OR (95% Cl)	< 0.001	1.104 (1.066–1.144)	Reference	3.860 (2.126–7.009)
Term late-onset HDP, N (%)	NA	NA	74 (4.2)	28 (10.4)
Model 1: Crude OR (95% Cl)	< 0.001	1.083 (1.057–1.109)	Reference	3.161 (1.996–5.005)
Model 2: Crude OR (95% Cl)	< 0.001	1.078 (1.051–1.105)	Reference	2.898 (1.810–4.641)
Model 3: Crude OR (95% Cl)	< 0.001	1.075 (1.048–1.103)	Reference	2.686 (1.661–4.344)

## Discussion

New findings

In this retrospective cohort study of a Japanese population, MAP in early pregnancy was associated with an increased risk of HDP across all gestational-age subtypes. Importantly, the magnitude of association demonstrated a graded pattern according to timing of onset, with the strongest association observed in early-onset HDP, followed by preterm late-onset HDP and term late-onset HDP. These associations remained significant after adjustment for maternal characteristics. The present findings suggest heterogeneity in the relationship between maternal hemodynamic status in early pregnancy and the timing of HDP onset.

Comparison with previous studies

Previous studies have consistently demonstrated that elevated MAP in early pregnancy is associated with an increased risk of HDP, particularly early-onset disease [8,11]. The present findings are generally consistent with these observations. However, most previous studies have classified HDP only into early- and late-onset disease. In contrast, the present study further subdivided late-onset HDP into preterm late-onset and term late-onset subtypes, allowing evaluation of the association according to more granular gestational age categories. Furthermore, the present findings extend previous observations derived mainly from non-Japanese populations to a Japanese cohort.

Proposed mechanisms

Elevated early pregnancy MAP may reflect increased maternal vascular resistance and impaired cardiovascular adaptation [11]. These changes may be associated with underlying pathophysiological processes that contribute to the risk of HDP, including suboptimal early placental development. The stronger association observed for early-onset HDP in this study may reflect a greater contribution of these mechanisms to earlier disease. Furthermore, the greater attenuation of the association after BMI adjustment in early-onset HDP suggests that maternal adiposity and related cardiometabolic factors may partly explain the stronger association in early-onset disease.

Implication and future direction

Early pregnancy MAP, measured at 10-13 weeks, may be a useful indicator for risk stratification of HDP, particularly for early-onset disease. In this study, MAP was analyzed both as a continuous variable and using a previously reported pragmatic threshold of ≥90 mmHg for exploratory stratified analysis [10,12]. Higher MAP was consistently associated with increased risk of HDP across all subtypes, with numerically higher odds ratios for early-onset disease. Although this threshold was not derived from the present dataset, these findings support the potential utility of early pregnancy MAP as an accessible tool for initial risk identification. Further studies using data-driven cut-off derivation and external validation are required to establish clinically actionable thresholds and to integrate MAP into existing prediction models.

Strengths and limitations

This study has several strengths: first, the classification of HDP by timing of onset (early-onset, preterm late-onset, and term late-onset) in examining their association with MAP in early pregnancy. This approach suggests potential differences in the association across onset timing and provides a perspective beyond the conventional dichotomous classification of early- and late-onset disease. Second, MAP is a readily obtainable parameter in routine clinical practice and does not require specialized measurements. Compared with existing prediction models that require uterine artery Doppler assessment and placental biomarkers, MAP may serve as a simple and accessible indicator for initial risk assessment. Third, this study was conducted in a Japanese population and extends previous findings, which have been mainly derived from non-Japanese populations, to a different population.

However, this study has several limitations. First, this was a single-center retrospective cohort study, and differences in blood pressure measurement devices (automated versus manual) were not distinguished; therefore, selection bias, information bias, and measurement error cannot be fully excluded. Secondly, MAP in early pregnancy was based on a single measurement. As demonstrated by MacMahon et al., blood pressure measured at a single time point is subject to substantial within-person variability due to biological fluctuation and measurement error, resulting in regression dilution bias [15]. Consequently, the observed association in this study between early pregnancy MAP and HDP is likely to be attenuated compared with the true underlying relationship. Third, residual confounding due to unmeasured variables may persist. Future prospective studies with repeated blood pressure measurements, more comprehensive adjustment for potential confounders, and validation in large-scale cohort studies are warranted to confirm and extend these findings.

## Conclusions

Higher MAP in early pregnancy was associated with an increased risk of HDP, with a stronger association observed for earlier-onset disease. These findings suggest that early pregnancy MAP may reflect maternal hemodynamic status related to the development and timing of HDP. Further studies are needed to confirm these associations and clarify their clinical implications.
